# Association of *ANKRD*55 Gene Polymorphism with HT: A Protective Factor for Disease Susceptibility

**DOI:** 10.1155/2022/7300796

**Published:** 2022-08-09

**Authors:** Yu-die Fang, Jing Zhao, Xin-juan Zhuang, Jian-bin Xu, Tian-tian Cai, Xiao-rong Yang, Kai-da Mu, Jin-an Zhang

**Affiliations:** ^1^Graduate School, Shanghai University of Traditional Chinese Medicine, Shanghai 201203, China; ^2^Department of Endocrinology & Rheumatology, Shanghai University of Medicine & Health Sciences Affiliated Zhoupu Hospital, Shanghai 201599, China; ^3^Department of Endocrinology, Jinshan Branch of Shanghai Sixth People's Hospital, Shanghai 201318, China; ^4^Shanghai University of Traditional Chinese Medicine, Shanghai 201203, China

## Abstract

**Purpose:**

Recent studies have shown that Ankyrin Repeat Domain 55 (*ANKRD55*) gene polymorphism is a risk factor for multiple autoimmune diseases, but its association with autoimmune thyroid diseases (AITDs) has not been reported. The purpose of this study was to investigate the potential relationship between polymorphism of the *ANKRD55* gene and AITDs.

**Methods:**

For this study, we enrolled 2050 subjects, consisting of 1220 patients with AITD and 830 healthy subjects. Five loci (rs321776, rs191205, rs7731626, rs415407, and rs159572) of the *ANKRD55* gene were genotyped using Multiplex PCR combined with high-throughput sequencing.

**Results:**

The results showed that the allele frequencies of rs7731626 and rs159572 loci in HT patients were lower than those in normal controls (*P*=0.048 and *P*=0.03, respectively). In different genetic model analyses, rs7731626 and rs159572 were also significantly correlated with HT in allele, dominant and additive models before and after age and sex adjustment. There were no differences in rs321776, rs191205, or rs415407 of the *ANKRD55* gene in allele frequency or genotype frequency between AITDs patients and controls.

**Conclusions:**

This study for the first time found that rs7731626 and rs159572 of *ANKRD55* were significantly correlated with HT, and individuals carrying the A allele at these two loci had a lower probability of developing HT.

## 1. Introduction

Graves' disease (GD) and Hashimoto's thyroiditis (HT) are two main types of autoimmune thyroid diseases (AITDs). AITDs are one of the most widespread autoimmune disorders, with an estimated prevalence of 5% [1]. One study revealed that the incidence of AITDs in women versus men is about 5 : 1 and that the severity varies from patient to patient [2]. The common pathological features of AITD are lymphocyte infiltration in thyroid tissue and thyroid autoantibodies in serum. The most prominent characteristics of GD are positive TRAb and clinical hyperthyroidism manifestations such as excessive sweating, irritability, and emasculation. HT is characterized by thyroid lymphocytic infiltration, positive serum TgAb, and TPOAb, with or without hypothyroidism. Clinically, early diagnosis is difficult to predict the severity of GD and HT. The thyroid function of some GD patients returns to normal after drug withdrawal, while in others thyroid dysfunction recurs after discontinuation of the drug. Among HT patients, some develop hypothyroidism early in the disease, while others maintain normal thyroid function throughout life. [3].

The pathogenesis of AITDs has not been fully revealed; however, numerous studies suggest that it is the complex interaction of multiple factors, with genetic and environmental factors, that plays a key role in the occurrence and development of the disease. In recent years, AITDs have been considered to be polygenic diseases in which multiple immune-related genes are involved in susceptibility to AITDs. Moreover, the genetic variation study of AITDs revealed plentiful gene SNPs associated with AITDs, such as CTLA-4 [4] and CD14 [5]. However, the results of existing genetic studies cannot fully explain the etiology of AITDs and are not sufficient for clinical application. Therefore, it is necessary to further explore the genetic factors of AITDs to find a possible etiological treatment.

Ankyrin repeat domain-containing protein 55 (*ANKRD55*) is a protein that facilitates protein-protein interactions. Based on human transcriptomic data, *ANKRD55* is expressed in the ovaries, testes, endometrium, CD4+T cells, and monocytes, the latter two of which may be more important in autoimmune diseases [6, 7]. The exact biological function of *ANKRD55* is unknown. However, GWAS data suggest that *ANKRD55* is a genetic predisposition factor for such autoimmune diseases as Crohn's disease [8], multiple sclerosis (MS) [7, 9], and rheumatoid arthritis (RA) [10–12].

For the typical autoimmune diseases, we designed a case-control study to study the relationship between the AITDs and *ANKRD55*, which may reveal some underlying mechanisms of AITDs.

## 2. Materials and Methods

### 2.1. Study Subjects

Between 2018 and 2020, 1220 AITDs patients and 830 healthy controls were enrolled at the Endocrinology Department, Shanghai University of Medicine & Health Sciences Affiliated Zhoupu Hospital. There were 753 GD patients (212 males and 541 females) and 467 HT individuals (71 males and 396 females) among the AITDs patients. The diagnostic criteria for patients with GD refer to the American Thyroid Association Guidelines for Diagnosis and Management of Hyperthyroidism and Other Causes of Thyrotoxicosis (2016) [13], including the clinical manifestations and biochemical indicators of hyperthyroidism. Graves' ophthalmopathy (GO) was diagnosed by the clinical assessment criteria from the Williams Textbook of Endocrinology. Patients with HT were predominantly TgAb or TPOAb positive, accompanied by euthyroidism or hypothyroidism. Patients with other chronic or congenital conditions were not included. Healthy controls who did not suffer from any chronic diseases were enrolled in the Health Check-up Center of the same hospital. The Ethics Committee of Shanghai University of Medicine & Health Sciences Affiliated Zhoupu Hospital approved this study, and all patients and controls signed written informed consent.

### 2.2. Genomic DNA Extraction and Genotyping

Peripheral venous samples (2 ml) were collected from all participants and genomic DNA was extracted using the Relax Gene Blood DNA System (Tiagen Biotechnology, Beijing, China). Subsequently, the concentration and quality of DNA were examined by NanoDrop 2000 Spectro-photometer (Thermo Scientific Company, Waltham, MA, USA). Shanghai Biowing Applied Biotechnology Company (https://www.biowing.com.cn/) utilized Multiplex PCR and high-throughput sequencing genotyping methods to detect five loci of the *ANKRD55* gene, namely, rs321776, rs191205, rs7731626, rs415407, and rs159572. Specific primers designed for the target site of this gene are shown in Table 1. In addition, in the SNPs detection process, a blank sample without DNA was used as a negative control, and a duplication of the same DNA sample was used as a positive control to ensure the quality of genotyping.

### 2.3. Statistical Analysis

Allele and genotype distribution of five SNPs between groups was evaluated using the Chi-square test and Fisher's exact test. Continuous variables were expressed as mean ± standard deviation, and categorical variables were expressed as frequency and percentage. Multivariate logistic regression analyses were performed to calculate *P* value and OR for confounding variables such as age and sex. Allelic equilibrium between samples was checked by Hardy-Weinberg equilibrium (HWE) and *P* > 0.05 was considered as valid data. SPSS statistical software (SPSS for Windows version 21.0, IBM SPSS Inc., USA) and SNPstats software (https://www.snpstats.net) were used for all analyses. Odds ratios were also estimated using the multiple inheritance model (Allele, dominant, recessive, homozygous, and additive) in SNPstats software. *P* < 0.05 was considered significant.

## 3. Results

### 3.1. Clinical Characteristics

Table 2 shows the clinical characteristics of all subjects in the control and patient groups, including 1220 patients with AITD (753 GD and 467 HT) and 830 normal controls. The gender distribution of each group was as follows: 283 males and 937 females in AITD patients, 212 males, and 541 females in GD patients, 71 males and 396 females in HT patients, and 329 males and 501 females in controls. The average age of the AITD group, GD group, HT group, and control group was 41.71 ± 14.20 years, 41.35 ± 14.45 years, 42.29 ± 13.78 years, and 38.77 ± 10.39 years, respectively. Among the AITD patients, 225 (18.44%) had a family history of thyroid disease. There were 456 cases (37.38%), 299 cases (24.51%), 416 cases (34.10%), and 49 cases (4.02%) of those without goiter, with I, II, and III degrees goiter. Among the GD patients, 108 (14.34%) had ophthalmopathy. Among the HT patients, 197 (42.18%) had hypothyroidism.

### 3.2. Association of the Five SNPs with AITD, GD, or HT

In this study, we performed the HWE test in the control group using SNPstats software, which showed that all SNPs conformed to HWE.  Table 3 shows allele and genotype frequencies at five loci in the AITD, GD, HT, and control groups. The frequencies of rs321776, rs191205, and rs415407 did not differ between disease groups and control groups (*P* > 0.05). Nevertheless, the allele distribution of rs7731626 and rs159572 was significantly different between the HT group and the control group. At rs7731626, the allele frequencies of A vs G were 8.35% vs 91.65% in the healthy control group, and 6.15% vs 93.85% in the HT group, respectively; the A allele frequency was significantly lower in the HT group than in the control group (*P*=0.048). At rs159572, the allele frequencies of A vs C were 34.25% vs 65.75% in the healthy controls, and 30.05% vs 69.95% in the HT group; the A allele frequency was significantly lower in the HT group than in the control group (*P*=0.03). No significant detectable difference was found in the genotype distribution of rs7731626 and rs159572 SNPs between the controls and AITDs, GD, or HT.

We also analyzed the correlation of the five SNPs of the *ANKRD55* gene with AITDs, GD, and HT in different genetical models. The results are shown in Table 4. Rs7731626 polymorphism was associated with HT under the allele, dominant, and additive models, and the ORs were OR = 0.70 (95% CI = 0.49–0.98, *P* = 0.04), OR = 0.65 (95% CI = 0.45–0.94, *P* = 0.02), and OR = 0.63 (95% CI = 0.43–0.91, *P* = 0.01), respectively. Rs159572 polymorphism and HT were correlated in allele, dominant and additive models, and the ORs were OR = 0.80 (95% CI = 0.67–0.97, *P* = 0.02), OR = 0.73 (95% CI = 0.57–0.93, *P* = 0.01), OR = 0.73 (95% CI = 0.57–0.95, *P* = 0.02). There was no significant correlation of HT with rs321776, rs191205, or rs415407 of *ANKRD55* before and after adjusting for age and gender in all genetic models. There was no significant difference between these five SNPs and AITDs or GD compared with the control group (Supplementary Table S1).

## 4. Discussion

The ankyrin protein repeat (ANK) was first discovered in 1987 as a protein consisting of 33 residue sequence motifs ubiquitous in living organisms [14, 15]. ANK is found in many functional proteins, such as cyclin-dependent kinase inhibitors, transcriptional regulators, cytoskeletal organizers, developmental regulators, membrane proteins, and toxins [16]. The *ANKRD55* gene, located on chromosome 5q11.2, is an ankyrin repeat domain (ARD) gene that encodes the ankyrin repeat domain protein 55, and its specific function is currently unknown. Transcriptome databases (https://www.amazonia.transcriptome.eu) show that *ANKRD55* is expressed on CD4+T cells and monocytes, suggesting its possible involvement in inflammatory or autoimmune diseases [6, 7, 17]. Protein interactomes identification and bioinformatics analysis showed that the *ANKRD55* protein was associated with cell cycle, nucleotide and ATP binding, and lipid and amino acid metabolism [18].

While the exact function of *ANKRD55* is unclear, there may be a link between genetic variants of *ANKRD55* and autoimmune diseases. The *ANKRD55* gene contains many SNPs that are associated with different autoimmune disease risks. The first SNP of *ANKRD55* (rs6859219) was initially identified as a risk factor in genome-wide association analysis of rheumatoid disease, and subsequent reports have stabilized this conclusion [10, 19, 20]. Rs7731626, which is located in dendritic cell-specific immune cell-enhancing regions [21], has also been reported as a contributor to RA in European populations [19]. Further studies have underscored pleiotropy of *ANKRD55* by linking many SNPs to multiple sclerosis [7], type 1 diabetes [22], Crohn's disease [20, 23], juvenile idiopathic arthritis [24], celiac disease [12], and inflammatory myopathies [17, 25], as well as post traumatic stress disorder [26], cognitive decline in Alzheimer's disease [27] and type 2 diabetes [28, 29]. Many of the diseases mentioned above are autoimmune diseases. To explore the link between *ANKRD55* and AITD, we selected five polymorphisms (rs321776, rs191205, rs7731626, rs415407, and rs159572) of *ANKRD55*. Among them, rs321776 is a missense mutation, and rs191205, rs7731626, rs415407, and rs159572 are within the intron region.

Our study investigated the relationship between five SNPs of *ANKRD55* in AITD, and only rs7731626 and rs159572 alleles and genotypes had an association with HT. The frequencies of allele A of rs7731626 and rs159572 were both lower than those of the control group, suggesting that these two loci may be involved in the development of HT and play a protective role. The genetic model is one of the common methods to analyze the association between different genotypes and diseases and is also the main method to determine how SNP affects the risk of AITD. In this genetic model analysis, allele, dominant and additive models of rs7731626 and rs159572 were significantly associated with HT before and after adjusting for age and sex. Taking the commonly used dominant model of rs7731626 as an example, compared with wild-type genotype GG, genotype AG + AA reduced the risk of HT by 35%(OR = 0.65). This genetic model indicated a single copy of A was enough to modify the risk.

*ANKRD55* can be induced after inflammatory stimulation, and its high expression levels have been shown to increase a patient's susceptibility to inflammation [6]. Lopez de Lapuente et al. isolated a variety of immune cells from human peripheral blood mononuclear cells and measured the expression level of the *ANKRD55* gene in them. The results showed that the *ANKRD55* transcript was specifically overexpressed in CD4+ T cells, but was not detected or rarely expressed in other subpopulations [6, 30]. In addition, increased protein expression of *ANKRD55* in mice with autoimmune encephalomyelitis uncovered that this molecule can be used as a disease biomarker [6, 30]. However, this study only suggests that *ANKRD55* exists in CD4+T cells, but the positive or negative effect of *ANKRD55* protein on T lymphocytes was not determined. HT is clinical comorbidity with other autoimmune diseases, that is, patients with HT can have multiple other autoimmune diseases, such as RA and MS. These two diseases have been reported to share the same pathogenic genes and risk loci with HT [31–33]. Similarly, our results showed that the A allele of rs7731626 was reduced in AITD, which is consistent with RA [19]and MS [34]. The Genotype-Tissue Expression (GTEx) Project lists the MS and RA lead risk variant rs7731626 as the SNP with the most significant cis-expression quantitative trait locus(cis-eQTL) effect in *ANKRD55* [19]. Subsequent reports showed that rs7731626 colocalized with CD4+ cell-specific eQTL [35]. It is suggested that this locus may affect the expression of *ANKRD55* and mediate immune regulation.

The IL6ST gene is near *ANKRD55*, and IL-6 is more attractive for its precise role in inflammation and autoimmune disease. IL-6 mediates antiinflammatory and proinflammatory responses through two different pathways [36, 37]. In MS, blocking IL-6 is thought to limit immune-mediated tissue damage. IL-6mAb has been shown to inhibit experimental autoimmune encephalomyelitis (EAE) [38]. Although it has been reported that there is no strong linkage imbalance (LD) between *ANKRD55* and IL-6ST [6, 39], some studies have proved that rs7731626 polymorphism is related to increased IL-6 expression in CD4+T cells [40]. In addition, increased methylation of the *ANKRD55-IL6ST* protective allele site CpG in CD4+T cells reduced the mRNA levels of IL6ST [41]. Thus, *ANKRD55* polymorphisms may mediate immune responses by affecting IL6.

In conclusion, we found that SNPs at rs7731626 and rs159572 of the *ANKRD55* gene were associated with HT susceptibility and that the A allele of these two loci was a protective factor of HT susceptibility.

## Figures and Tables

**Table 1:** Sequence of primers used for five SNPs in *ANKRD55* gene.

SNPs	Primer	
rs321776	Forward	-GTGGTGATGATGTCATTGACTTC
	Reverse	-CCCTGCTCTTTTATATTCGCATAG

rs191205	Forward	-TTTGTTTGCCCAGTTTAGAACC
	Reverse	-TGGTTATTTATAAGCCGTCACAAG

rs7731626	Forward	-AGGTTTCATGTTTCAGAACTGTAC
	Reverse	-AAGCATCTGGAATTGTTTACTGAC

rs415407	Forward	-TCCCACTTTTAAGTATTTTGCTGG
	Reverse	-TATCAGCAGTCTGTTCTTTTTCAC

rs159572	Forward	-CTAGAATTGTTGGTAGCACATCTG
	Reverse	-TACAGTGAGCTCTATGTTAAGTGC

**Table 2:** Clinical features of subjects including AITD patients and controls.

Items	AITD	GD	HT	Controls
Number	1220	753	467	830

Gender				
Male	283 (23.20%)	212 (28.15%)	71 (15.20%)	329 (39.64%)
Female	937 (76.80%)	541 (71.85%)	396 (84.80%)	501 (60.36%)
Age (years)	41.71 ± 14.20	41.35 ± 14.45	42.29 ± 13.78	38.77 ± 10.39

Family history				
(+)	225 (18.44%)	147 (19.52%)	78 (16.70%)	—
(−)	995 (81.56%)	606 (80.48%)	389 (83.30%)	—

Ophthalmopathy				
(+)	—	108 (14.34%)	—	—
(−)	—	645 (85.66%)	—	—

Hypothyroidism				
(+)	—	—	197 (42.18%)	—
(−)	—	—	270 (57.82%)	—

Goiter				
No goiter	456 (37.38%)	223 (29.61%)	233 (49.89%)	—
Degree I	299 (24.51%)	193 (25.63%)	106 (22.70%)	—
Degree II	416 (34.10%)	296 (39.31%)	120 (25.70%)	—
Degree III	49 (4.02%)	41 (5.44%)	8 (1.71%)	—

AITD, autoimmune thyroid diseases; GD, graves' disease; HT, hashimoto's thyroiditis.

**Table 3:** Allele and genotype frequencies of the five loci in AITD, GD, HT, and controls.

Gene/SNP	Controls	AITD	*P* value (OR, 95% CI)	GD	*P* value (OR, 95% CI)	HT	*P* value (OR, 95% CI)
ANKRD55	*n* (%)	*n* (%)	AITD vs. controls	*n* (%)	GD vs. controls	*n* (%)	HT vs. controls
rs321776							
C	1168 (71.39)	1561 (69.63)	0.23 (1.09, 0.95–1.25)	949 (68.47)	0.08 (1.15, 0.98–1.34)	612 (71.50)	0.96 (0.99, 0.8–1.20)
T	468 (28.61)	681 (30.37)		437 (31.53)		244 (28.50)	
TT	62 (7.58)	92 (8.21)	0.44	67 (9.67)	0.2	25 (5.84)	0.35
TC	344 (42.05)	497 (44.33)		303 (43.72)		194 (45.33)	
CC	412 (50.37)	532 (47.46)		323 (46.61)		209 (48.83)	

rs191205							
A	1172 (71.55)	1572 (69.99)	0.29 (1.08, 0.94–1.24)	961 (68.94)	0.12 (1.13, 0.97–1.33)	611 (71.71)	0.93 (0.99, 0.83–1.19)
G	466 (28.45)	674 (30.01)		433 (31.06)		241 (28.29)	
AA	414 (50.55)	540 (48.09)	0.55	331 (47.49)	0.24	209 (49.06)	0.33
AG	344 (42.00)	492 (43.81)		299 (42.90)		193 (45.31)	
GG	61 (7.45)	91 (8.10)		67 (9.61)		24 (5.63)	

rs7731626							
G	1503 (91.65)	2074 (91.85)	0.82 (0.97, 0.77–1.23)	1265 (90.62)	0.32 (1.14, 0.88–1.46)	809 (93.85)	0.048 (0.72, 0.52–1.00)
A	137 (8.35)	184 (8.15)		131 (9.38)		53 (6.15)	
AA	5 (0.61)	7 (0.62)	0.97	4 (0.57)	0.54	3 (0.70)	0.08
AG	127 (15.49)	170 (15.06)		123 (17.62)		47 (10.90)	
GG	688 (83.90)	952 (84.32)		571 (81.81)		381 (88.40)	

rs415407							
A	1131 (69.39)	1596 (71.57)	0.14 (0.90, 0.78–1.04)	975 (71.06)	0.32 (0.92, 0.79–1.08)	621 (72.37)	0.12 (0.87, 0.72–1.04)
C	499 (30.61)	634 (28.43)		397 (28.94)		237 (27.63)	
AA	391 (47.98)	565 (50.68)	0.30	346 (50.44)	0.60	219 (51.05)	0.17
AC	349 (42.82)	466 (41.79)		283 (41.25)		183 (42.66)	
CC	75 (9.20)	84 (7.53)		57 (8.31)		27 (6.29)	

rs159572							
C	1073 (65.75)	1491 (66.03)	0.85 (0.99, 0.86–1.13)	888 (63.61)	0.22 (1.10, 0.95–1.28)	603 (69.95)	0.03 (0.82, 0.69–0.99)
A	599 (34.25)	767 (33.97)		508 (36.38)		259 (30.05)	
AA	94 (11.52)	127 (11.25)	0.98	85 (12.18)	0.36	42 (9.75)	0.08
AC	371 (45.47)	513 (45.44)		338 (48.42)		175 (40.60)	
CC	351 (43.01)	489 (43.31)		275 (39.40)		214 (49.65)	

AITD, autoimmune thyroid diseases; GD, graves' disease; HT, hashimoto's thyroiditis. 95% CI, 95% confidence interval; OR, odds ratio.

**Table 4:** Odds ratios (ORs) of the associations of five polymorphisms in the *ANKRD55* gene with HT before and after adjusting for age and gender.

Comparison models	Unadjusted		Adjusted	
	OR (95% CI)	*P* value	OR (95% CI)	*P* value
rs321776				
Allele model	0.99 (0.82–1.20)	0.96	0.99 (0.81–1.20)	0.88
Dominant model	1.06 (0.84–1.34)	0.61	1.06 (0.83–1.35)	0.64
Recessive model	0.76 (0.47–1.22)	0.25	0.72 (0.44–1.19)	0.19
Homozygous model	0.79 (0.49–1.30)	0.36	0.76 (0.45–1.27)	0.29
Additive model	1.11 (0.87–1.42)	0.39	1.12 (0.87–1.44)	0.40

rs191205				
Allele model	0.99 (0.82–1.20)	0.93	0.99 (0.81–1.20)	0.89
Dominant model	1.06 (0.84–1.34)	0.62	1.06 (0.83–1.35)	0.64
Recessive model	0.74 (0.46–1.21)	0.22	0.72 (0.44–1.20)	0.20
Homozygous model	0.78 (0.47–1.29)	0.33	0.76 (0.45–1.28)	0.30
Additive model	1.11 (0.87–1.42)	0.39	1.11 (0.86–1.43)	0.41

rs7731626				
Allele model	0.72 (0.52–1.00)	<0.05	0.70 (0.49–0.98)	0.04
Dominant model	0.68 (0.48–0.97)	0.03	0.65 (0.45–0.94)	0.02
Recessive model	1.14 (0.27–4.80)	0.86	1.67 (0.36–7.80)	0.52
Homozygous model	1.08 (0.26–4.56)	0.91	1.57 (0.34–7.38)	0.57
Additive model	0.67 (0.47–0.96)	0.03	0.63 (0.43–0.91)	0.01

rs415407				
Allele model	0.86 (0.72–1.04)	0.11	0.90 (0.74–1.09)	0.28
Dominant model	0.88 (0.70–1.12)	0.30	0.92 (0.72–1.17)	0.49
Recessive model	0.66 (0.42–1.05)	0.07	0.74 (0.46–1.19)	0.20
Homozygous model	0.64 (0.40–1.03)	0.64	0.72 (0.44–1.18)	0.19
Additive model	0.94 (0.73–1.19)	0.60	0.96 (0.74–1.23)	0.73

rs159572				
Allele model	0.83 (0.69–0.99)	0.03	0.80 (0.67–0.97)	0.02
Dominant model	0.77 (0.61–0.97)	0.03	0.73 (0.57–0.93)	0.01
Recessive model	0.83 (0.56–1.22)	0.34	0.83 (0.55–0.23)	0.34
Homozygous model	0.73 (0.49–1.09)	0.13	0.71 (0.47–1.08)	0.11
Additive model	0.77 (0.60–0.99)	0.04	0.73 (0.57–0.95)	0.02

Allele model = G vs. C; dominant model = (GG + GC) vs. CC; recessive model = GG vs. (GC + CC); homozygous model = GG vs. CC; additive model = GC vs. CC 95% CI, 95% confidence interval; OR, odds ratio.

## Data Availability

The data that support the findings of this study are available from the corresponding author upon reasonable request.

## Supplementary Materials

Supplementary Materials Table S1: Odds ratios (ORs) of the association of five polymorphisms of the *ANKRD55* gene with AITD and GD before and after adjusting for age and gender.
