# Supplementary material for: Association of ANKRD55 Gene Polymorphism with HT: A Protective Factor for Disease Susceptibility
Source: Int J Endocrinol. 2022 Aug 9;2022:7300796. doi: 10.1155/2022/7300796 (PMC9381225; doi:10.1155/2022/7300796)
Supplement: Supplementary Materials — Table S1: Odds ratios (ORs) of the association of five polymorphisms of the ANKRD55 gene with AITD and GD before and after adjusting for age and gender. [file 7300796.f1.docx]

**Supplementary material**

**Table S1：Odds ratios (ORs) of the association of five polymorphisms of ANKRD55 gene with AITD and GD before and after adjusting for age and gender**

| Comparison models | AITD | | | | | GD | | | |
| --- | --- | --- | --- | --- | --- | --- | --- | --- | --- |
|  | | Unadjusted | | Adjusted | | Unadjusted | | Adjusted | |
|  | | OR(95%CI) | *P* value | OR(95%CI) | *P* value | OR(95%CI) | *P* value | OR(95%CI) | *P* value |
| rs321776 | |  |  |  |  |  |  |  |  |
| Allele model | | 1.15(0.98-1.35) | 0.08 | 1.15(0.98-1.34) | 0.10 | 1.09 (0.95-1.26) | 0.22 | 1.09 (0.94-1.26) | 0.25 |
| Dominant model | | 1.16 (0.95-1.42) | 0.15 | 1.16 (0.94-1.42) | 0.16 | 1.12 (0.94-1.35) | 0.21 | 1.12 (0.93-1.35) | 0.23 |
| Recessive model | | 1.31 (0.91-1.87) | 0.15 | 1.28 (0.89-1.85) | 0.18 | 1.09 (0.78-1.52) | 0.61 | 1.08 (0.77-1.52) | 0.65 |
| Homozygous model | | 1.38 (0.95-2.01) | 0.09 | 1.35 (0.93-1.98) | 0.12 | 1.15 (0.81-1.63) | 0.43 | 1.14 (0.80-1.62) | 0.47 |
| Additive model | | 1.12 (0.91-1.39) | 0.28 | 1.12 (0.90-1.39) | 0.30 | 1.12 (0.93-1.35) | 0.24 | 1.12 (0.92-1.35) | 0.26 |
| rs191205 | |  |  |  |  |  |  |  |  |
| Allele model | | 1.14(0.97-1.33) | 0.11 | 1.13(0.96-1.32) | 0.14 | 1.08 (0.94-1.25) | 0.28 | 1.08 (0.93-1.25) | 0.31 |
| Dominant model | | 1.13 (0.92-1.38) | 0.23 | 1.12 (0.91-1.38) | 0.27 | 1.10 (0.92-1.32) | 0.28 | 1.10 (0.91-1.32) | 0.32 |
| Recessive model | | 1.32 (0.92-1.90) | 0.13 | 1.30 (0.90-1.87) | 0.17 | 1.10 (0.78-1.54) | 0.59 | 1.10 (0.78-1.55) | 0.60 |
| Homozygous model | | 1.37 (0.94-2.00) | 0.10 | 1.34 (0.92-1.97) | 0.13 | 1.14 (0.81-1.62) | 0.45 | 1.14 (0.80-1.63) | 0.47 |
| Additive model | | 1.09 (0.88-1.34) | 0.24 | 1.08 (0.87-1.34) | 0.47 | 1.10 (0.91-1.32) | 0.34 | 1.09 (0.90-1.32) | 0.38 |
| rs7731626 | |  |  |  |  |  |  |  |  |
| Allele model | | 1.14(0.88-1.47) | 0.31 | 1.13(0.87-1.46) | 0.36 | 0.97 (0.77-1.23) | 0.82 | 0.95 (0.75-1.20) | 0.65 |
| Dominant model | | 1.16 (0.89-1.52) | 0.28 | 1.14 (0.87-1.49) | 0.35 | 0.97 (0.76-1.24) | 0.80 | 0.93 (0.72-1.20) | 0.58 |
| Recessive model | | 0.94 (0.25-3.51) | 0.93 | 1.13 (0.29-4.36) | 0.86 | 1.02 (0.32-3.22) | 0.98 | 1.24 (0.38-4.08) | 0.72 |
| Homozygous model | | 0.96 (0.26-3.61) | 0.61 | 1.16 (0.30-4.45) | 0.83 | 1.01 (0.32-3.20) | 0.98 | 1.22 (0.37-4.04) | 0.74 |
| Additive model | | 1.17 (0.89-1.53) | 0.27 | 1.14 (0.86-1.50) | 0.36 | 0.97 (0.75-1.24) | 0.80 | 0.92 (0.71-1.19) | 0.53 |
| rs415407 | |  |  |  |  |  |  |  |  |
| Allele model | | 0.92(0.79-1.08) | 0.32 | 0.91(0.78-1.07) | 0.26 | 0.90 (0.78-1.03) | 0.14 | 0.90 (0.78-1.04) | 0.16 |
| Dominant model | | 0.91 (0.74-1.11) | 0.34 | 0.90 (0.73-1.10) | 0.29 | 0.90 (0.75-1.08) | 0.24 | 0.89 (0.74-1.08) | 0.24 |
| Recessive model | | 0.89 (0.62-1.28) | 0.54 | 0.88 (0.61-1.27) | 0.49 | 0.80 (0.58-1.11) | 0.19 | 0.82 (0.59-1.15) | 0.25 |
| Homozygous model | | 0.86 (0.59-1.25) | 0.42 | 0.84 (0.58-1.23) | 0.37 | 0.78 (0.55-1.09) | 0.14 | 0.79 (0.56-1.12) | 0.18 |
| Additive model | | 0.92 (0.74-1.13) | 0.42 | 0.91 (0.73-1.13) | 0.38 | 0.92 (0.76-1.12) | 0.41 | 0.92 (0.76-1.11) | 0.38 |
| rs159572 | |  |  |  |  |  |  |  |  |
| Allele model | | 1.10(0.95-1.28) | 0.21 | 1.11(0.95-1.29) | 0.20 | 0.99 (0.86-1.13) | 0.85 | 1.00 (0.87-1.14) | 0.95 |
| Dominant model | | 1.16 (0.95-1.43) | 0.15 | 1.16 (0.95-1.43) | 0.15 | 0.99 (0.82-1.18) | 0.90 | 1.00 (0.83-1.20) | 0.96 |
| Recessive model | | 1.07 (0.78-1.46) | 0.69 | 1.08 (0.79-1.48 | 0.63 | 0.97 (0.73-1.29) | 0.85 | 0.99 (0.74-1.32) | 0.94 |
| Homozygous model | | 1.15 (0.83-1.61) | 0.40 | 1.17 (0.84-1.64) | 0.36 | 0.97 (0.72-1.31) | 0.84 | 0.99 (0.73-1.34) | 0.94 |
| Additive model | | 1.16 (0.94-1.44) | 0.17 | 1.16 (0.93-1.45) | 0.18 | 0.99 (0.82-1.20) | 0.94 | 1.00 (0.82-1.21) | 0.98 |

Allele model = G vs. C; Dominant model = (GG+GC) vs. CC; Recessive model = GG vs. (GC+CC); Homozygous model = GG vs. CC; Additive model = GC vs. CC

95% CI, 95% confidence interval; OR, odds ratio.
